# *Astragalus mongholicus* and *Scutellaria baicalensis* Extracts Mixture Target Pyroptosis in Ischemic Stroke via the NLRP3 Pathway

**DOI:** 10.3390/ijms26020501

**Published:** 2025-01-09

**Authors:** Geon Ko, Jinho Kim, Yongjae Hong, Yeong-Jae Jeon, Hyun-Man Baek, Donghun Lee, Keun-A Chang

**Affiliations:** 1 Department of Health Sciences and Technology, Gachon Advanced Institute for Health Science & Technology, Gachon University, Incheon 21999, Republic of Korea; sirius9725@gachon.ac.kr (G.K.); qpdlzhfl@gachon.ac.kr (J.K.); michael369@gachon.ac.kr (Y.H.); yeong@gachon.ac.kr (Y.-J.J.); hmbaek98@gachon.ac.kr (H.-M.B.); 2 Department of Pharmacology, College of Medicine, Gachon University, Incheon 21999, Republic of Korea; 3 Department of Molecular Medicine, College of Medicine, Gachon University, Incheon 21999, Republic of Korea; 4 Department of Basic Neuroscience, Neuroscience Research Institute, Gachon University, Incheon 21999, Republic of Korea; 5 Department of Herbal Pharmacology, College of Korean Medicine, Gachon University, Seongnam 13120, Republic of Korea

**Keywords:** *Astragalus mongholicus*, EAHM, ischemic stroke, inflammation, middle cerebral artery occlusion, pyroptosis, *Scutellaria baicalensis*

## Abstract

Ischemic stroke, caused by blocked cerebral blood flow, requires prompt intervention to prevent severe motor and cognitive impairments. Despite extensive drug development efforts, the failure rate of clinical trials remains high, highlighting the need for novel therapeutic approaches. This study investigated the therapeutic potential of a natural herbal extract mixture of *Astragalus mongholicus* Bunge (AM) and *Scutellaria baicalensis* Georgi (SB), traditionally used in Eastern Asian herbal medicine (EAHM) for ischemic stroke treatment. Using transient middle cerebral artery occlusion (tMCAO) and photothrombotic (PTB) mouse models, oral administration of the AM-SB mixture was evaluated during both acute and chronic phases. Results showed that AM-SB significantly reduced infarction volume, inflammation (IL-1β, TNF-α), and pyroptosis-related markers (NLRP3, GSDMD, ASC, Caspase-1), while decreasing gliosis and improving cerebral metabolites. Behavioral assessments revealed that early and sustained AM-SB intervention enhanced motor and cognitive functions, as measured by mNSS, Rotarod, Novel Object Recognition, and Passive Avoidance tests. These findings suggest that AM-SB extract is a promising alternative therapy for ischemic stroke management.

## 1. Introduction

Stroke is one of the leading causes of death and disability worldwide, with ischemic stroke accounting for a significant proportion of cases [1]. Its incidence continues to rise, while post-stroke sequelae remain common, resulting in an increasing number of patients requiring rehabilitation therapy. This places a growing burden on healthcare systems and leads to escalating societal costs [2]. Ischemic stroke occurs when a blockage in cerebral blood vessels restricts blood flow, leading to symptoms such as limb weakness or numbness, impaired sensation, slurred speech, dizziness, visual disturbances, and severe headaches [3]. Without timely intervention, these symptoms worsen due to a cascade of molecular and cellular events following the ischemic insult, ultimately causing irreversible brain damage [4,5,6]. The affected brain regions include the ischemic core and the surrounding penumbra. The core is the central region where blood flow is critically reduced, leading to rapid and irreversible cell death [7]. The penumbra, however, retains some blood flow, leaving the tissue structurally intact but nonfunctional. This region represents a key therapeutic target as it holds the potential for recovery if blood flow is restored promptly [7,8,9,10,11]. Pyroptosis, a form of inflammatory programmed cell death, plays a significant role in the penumbra, exacerbating inflammation and contributing to secondary brain injury. This process involves inflammasome activation, Caspase-1 activation, and GSDMD-mediated pore formation, which result in the release of proinflammatory cytokines [12].

The primary treatment of ischemic stroke is the thrombolytic agent tissue plasminogen activator (tPA). tPA converts plasminogen to plasmin, dissolving blood clots, reopening occluded vessels, and restoring oxygen and nutrient delivery to ischemic brain tissue. When administered within 4.5 h of symptom onset, tPA significantly improves neurological recovery and survival rates. However, tPA has a narrow therapeutic time window, beyond which its efficacy declines while the risk of intracerebral hemorrhage increases. Furthermore, it is contraindicated in patients with conditions such as hypertension, a history of intracranial hemorrhage, anticoagulant use, or active bleeding disorders [13].

Given these limitations, alternative or complementary therapies are urgently needed. This study explores the potential of two widely used herbal medicines, *Astragalus mongholicus* Bunge (AM) and *Scutellaria baicalensis* Georgi (SB). AM, a primary component of BYHW Tang in East Asian herbal medicine (EAHM), has demonstrated significant efficacy in stroke treatment [14]. Its therapeutic effects are attributed to active components such as flavonoids and saponins, which exhibit antioxidant, anti-inflammatory, and neuroprotective properties. These components contribute to cholesterol reduction, improved blood circulation, and the prevention of atherosclerosis, thereby offering protection against cardiovascular diseases [15,16,17,18,19]. SB, traditionally used for neurological and cardiovascular disorders [20,21], contains baicalein, a flavonoid known for its anti-inflammatory and anti-apoptotic effects [22]. Recognizing their complementary mechanisms of action, this study aimed to evaluate the efficacy of an AM-SB mixture in preventing the progression of cerebral infarction and mitigating post-stroke sequelae. To investigate these effects, we utilized mouse models representing the acute, sub-chronic, and chronic phases of stroke. The photothrombotic (PTB) model was employed to replicate the acute phase, characterized by thrombus formation and vascular occlusion within 24 h, closely resembling the early stroke onset in clinical settings [23]. The transient middle cerebral artery occlusion (tMCAO) model was used to examine molecular and behavioral outcomes of AM-SB administration during the sub-chronic phase and evaluate its long-term effects on post-stroke sequelae.

## 2. Results

### 2.1. Effects of Combined AM-SB Extract Treatment on the PTB Stroke Mouse Model

**Figure 1:**
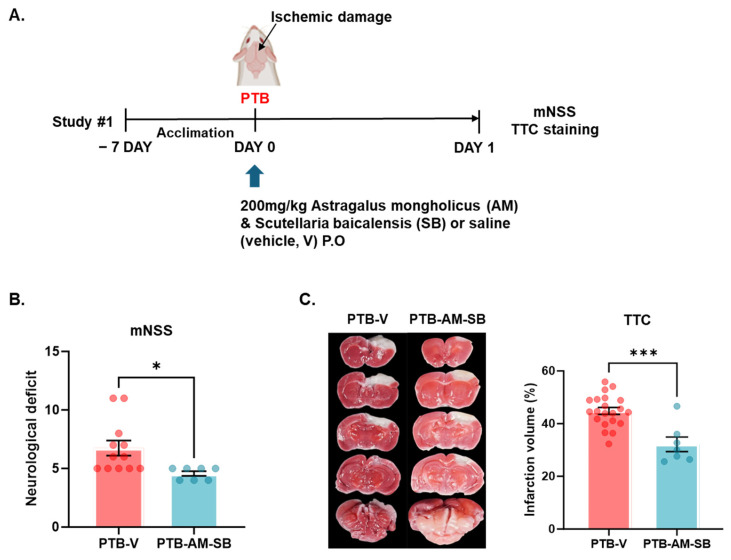
AM –SB alleviated ischemic injury in the PTB mouse brain. (**A**) Schematic overview of the experimental design. (**B**) Neurological deficit was evaluated with mNSS between PTB –V and PTB –AM –SB groups (PTB –V, n = 12; PTB –AM-SB, n = 7). (**C**) Representative TTC –stained brain sections and quantification of the infarction volumes calculated from TTC staining (PTB –V, n = 21; PTB –AM –SB, n = 7). Values are expressed as the mean ± SEM. Statistical analysis was performed using unpaired *t*-tests, * *p* < 0.05, *** *p* < 0.001.

In a PTB stroke mouse model, ischemic lesions caused by localized thrombosis appeared in the sensorimotor cortex of the ICR mice 24 h after photostimulation. Following the establishment of the PTB stroke model, we investigated the neuroprotective effects of AM-SB extract using mNSS and TTC staining (Figure 1A). The extent of neurological deficit was evaluated between the PTB-V and PTB-AM-SB groups using the mNSS and confirmed that AM-SB treatment reduced neurological deficits in the PTB group (PTB-V, 6.75 ± 0.6411; PTB-AM-SB, 4.571 ± 0.202, * *p* < 0.05) (Figure 1B).

The infarct volume was normalized to that of the control group to enable comparisons across treatment groups. The AM-SB extract combination treatment significantly reduced the infarct volume compared to the control group (PTB-V, 44.83 ± 1.313%; PTB-AM-SB, 32.15 ± 2.781%, *** *p* < 0.001) (Figure 1B). The AM-SB treatment demonstrated a pronounced neuroprotective effect, effectively reducing ischemic damage in the PTB stroke model. The synergistic effect of the combined AM-SB extract was evaluated in a PTB stroke mouse model, in which brain infarction was induced through photochemical activation of a photosensitive dye.

### 2.2. AM-SB Administration Alleviated Cerebral Ischemia/Reperfusion Injury and Restored Cerebral Metabolites in the Brains of tMCAO Mice

**Figure 2:**
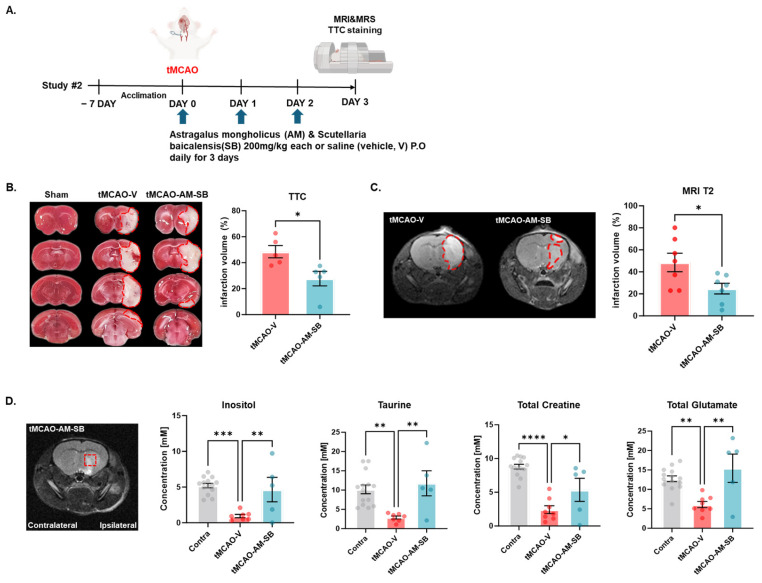
AM-SB alleviated ischemic injury and restored cerebral metabolites in the brains of tMCAO model mice. (**A**) Schematic overview of the experimental design. (**B**) Representative TTC-stained brain sections and quantification of infarction volumes (red dotted area) from TTC staining (tMCAO-V, n = 5; tMCAO-AM-SB, n = 5). (**C**) MRI images showing infarct regions and quantification of infarction volumes (red dotted area) from MRI T2 imaging (tMCAO-V, n = 7; tMCAO-AM-SB, n = 7). (**D**) Metabolite quantification using MRS in ROI (red dotted box) of tMCAO model mouse brain on day 3 post-tMCAO (Contra, n = 14; tMCAO-V, n = 7–8; tMCAO-AM-SB, n = 5). Values are expressed as the mean ± SEM. Statistical analysis was performed using the unpaired *t*-test for infarction volume, and one-way ANOVA followed by Tukey’s multiple comparisons test for metabolites. * *p* < 0.05, ** *p* < 0.01, *** *p* < 0.001, **** *p* < 0.0001.

To evaluate the neuroprotective effects of the herbal extract combination AM-SB against acute brain injury following ischemia and reperfusion (I/R), cerebral infarction was assessed 3 days following tMCAO (Figure 2A). tMCAO mice treated daily with 200 mg/kg of AM-SB (tMCAO-AM-SB) exhibited a significant reduction in the infarct volume compared to vehicle-treated tMCAO mice (tMCAO-V), based on the results of TTC staining (Figure 2B). Specifically, the infarction volume in the AM-SB treated tMCAO group (19.85 ± 7.775, ** *p* < 0.01) was significantly lower than that in tMCAO-V group (48.19 ± 4.451) (Figure 2B).

Furthermore, MRI analysis conducted 3 days post-stroke revealed a significantly lower infarction ratio in the penumbra area (highlighted by red dotted lines) in the tMCAO-AM-SB group (24.81 ± 4.834, * *p* < 0.05) compared to the tMCAO-V group (48.54 ± 8.378) (Figure 2C). In addition to these structural assessments, MRS analysis revealed significant differences in brain metabolite levels between the contralateral, vehicle, and AM-SB-treated tMCAO groups. In the AM-SB-treated group, the levels of inositol (Ins), taurine (Tau), total creatine (tCr), and glutamate (Glx) were significantly higher, and Acetylaspartate + N-Acetylaspartylglutamate (tNAA), glycerol phosphocholine + phosphatidylcholine (tCho), and lactate (Lac) all showed higher trends than in the tMCAO-V group.

The measured levels were as follows: Ins (contralateral: 5.204 ± 0.3087; AM-SB: 4.643 ± 1.709; vehicle: 0.9429 ± 0.2308); Tau (contralateral: 10.19 ± 1.140; AM-SB: 11.76 ± 3.245; vehicle: 2.886 ± 0.4032); tNAA (contralateral: 8.147 ± 0.5052; AM-SB: 4.704 ± 1.656; vehicle: 2.088 ± 0.5518); tCr (contralateral: 8.789 ± 0.3571; AM-SB: 5.357 ± 1.713; vehicle: 2.425 ± 0.5830); Glx (contralateral: 12.72 ± 0.7435; AM-SB: 15.44 ± 3.635; vehicle: 6.100 ± 0.7881); tCho (contralateral: 1.191 ± 0.06527; AM-SB: 1.089 ± 0.3196; vehicle: 0.6125 ± 0.09717); Lac (contralateral: 3.100 ± 0.5323; AM-SB: 11.15 ± 2.991; vehicle: 6.875 ± 0.9546) (Figure 2D and Figure S3). These findings indicate that AM-SB treatment effectively mitigates brain damage and restores metabolic balance following ischemic stroke in a tMCAO mouse model.

### 2.3. AM-SB Alleviated Gliosis and Inflammation in the Brains of tMCAO Mice

**Figure 3:**
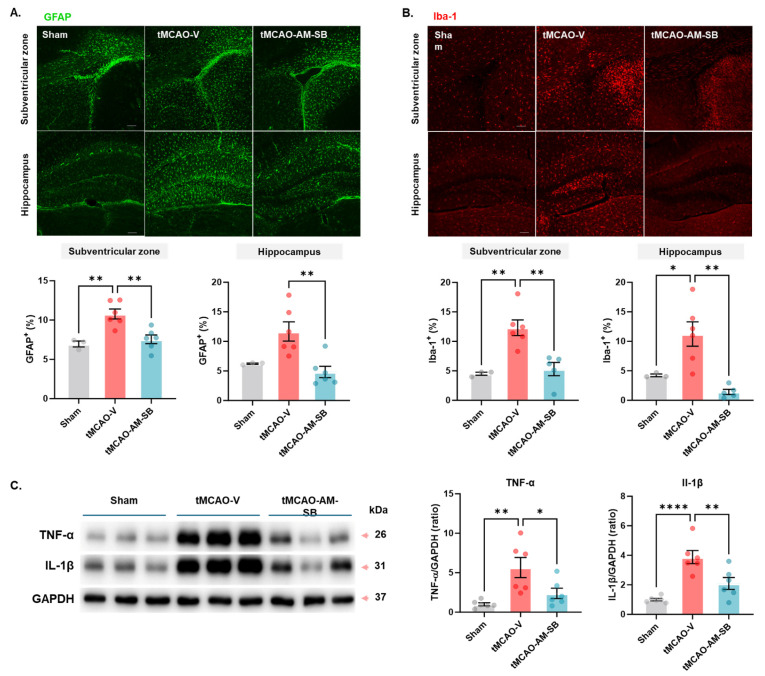
AM-SB reduced gliosis and inflammation in the brains of tMCAO model mice. Representative immunohistochemical images at 100× magnification and quantification of (**A**) GFAP and (**B**) Iba-1 staining in the cerebral tissue of tMCAO mice on day 3 post-tMCAO (sham, n = 3; tMCAO-V, n = 6; tMCAO-AM-SB, n = 5–6). (**C**) Representative Western blots showing the protein levels of TNF-α and IL-1β in the brains of tMCAO mice on day 3 (sham, n = 6; tMCAO-V, n = 6; tMCAO-AM-SB, n = 6). Values are expressed as the mean ± SEM. Statistical analysis was performed using one-way ANOVA followed by Tukey’s multiple comparisons test for GFAP and Iba-1, and Dunnett’s multiple comparisons test for Western blot. * *p* < 0.05, ** *p* < 0.01, **** *p* < 0.0001.

Inflammation plays a critical role in neural cell death during both the acute and chronic phases of cerebral ischemia [24,25]. AM-SB treatment was shown to reduce gliosis in the penumbra area following ischemic injury. We quantified the number of GFAP+ astrocytes and Iba1+ microglia 3 days post-tMCAO (Figure 3A,B and Figure S4). In the hippocampal penumbra, the tMCAO-AM-SB group showed a significant reduction in the number of GFAP+ astrocytes (4.834 ± 0.9611) compared to the tMCAO-V group (11.68 ± 1.619) (Figure 3A,B). Similarly, in the striatal penumbra, the number of GFAP+ astrocytes was significantly reduced in the tMCAO-AM-SB group (7.552 ± 0.5490, ** *p* < 0.01) compared to the tMCAO-V group (10.78 ± 0.6302) (Figure 3A,B). Moreover, the number of Iba1+ microglia, which serve as markers of activated microglia and indicate inflammation, was significantly lower in the hippocampal penumbra of the tMCAO-AM-SB group (1.422 ± 0.4417, ** *p* < 0.01) compared to the tMCAO-V group (11.25 ± 2.067) (Figure 3A,B). Similarly, in the striatal penumbra, the number of Iba1+ microglia was significantly reduced in the tMCAO-AM-SB group (5.303 ± 1.130, ** *p* < 0.01) compared to the tMCAO-V group (12.32 ± 1.326) (Figure 3A,B).

To further quantify the levels of inflammation, Western blots (WBs) were performed to measure the levels of inflammatory cytokines in the penumbra. The level of TNF-α was significantly lower in the tMCAO-AM-SB group (2.388 ± 0.6619, * *p* < 0.05) than in the tMCAO-V group (5.657 ± 1.281). The level of IL-1β was significantly lower in the tMCAO-AM-SB group (2.092 ± 0.4046, ** *p* < 0.01) compared to the tMCAO-V group (3.879 ± 0.4421) (Figure 3C and Figure S5). Overall, these results indicated that AM-SB treatment substantially reduced gliosis and inflammation in the brain following ischemic stroke.

### 2.4. AM-SB Alleviated Pyroptosis in the Brains of tMCAO Mice

Ischemia/reperfusion (I/R) injury induced by tMCAO leads to cellular organelle dysfunction, which contributes to inflammation and programmed cell death, particularly pyroptosis [12,26,27]. Pyroptosis is a distinct form of programmed cell death characterized by its inflammatory nature, differing from apoptosis and necroptosis. This process is mediated by the activation of inflammatory caspases, which cleave gasdermin proteins to form membrane pores. These pores lead to cell lysis and the release of proinflammatory cytokines, playing a critical role in the immune response and contributing to various diseases. Key biomarkers of pyroptosis include nucleotide-binding domain, leucine-rich repeat, and pyrin domain-containing 3 (NLRP3), gasdermin D (GSDMD), apoptosis-associated speck-like protein containing a CARD (ASC), Cleaved Caspase-1, TNF-α and IL-1β. NLRP3 and ASC are pivotal in activating Caspase-1, which subsequently drives pyroptosis and cytokine maturation. Activated Caspase-1 cleaves GSDMD, enabling it to membrane pores, while TNF-alpha and IL-1β further amplify the inflammatory response [12,28,29,30]. In this study, we evaluated these key pyroptosis markers using Western blot analysis.

**Figure 4:**
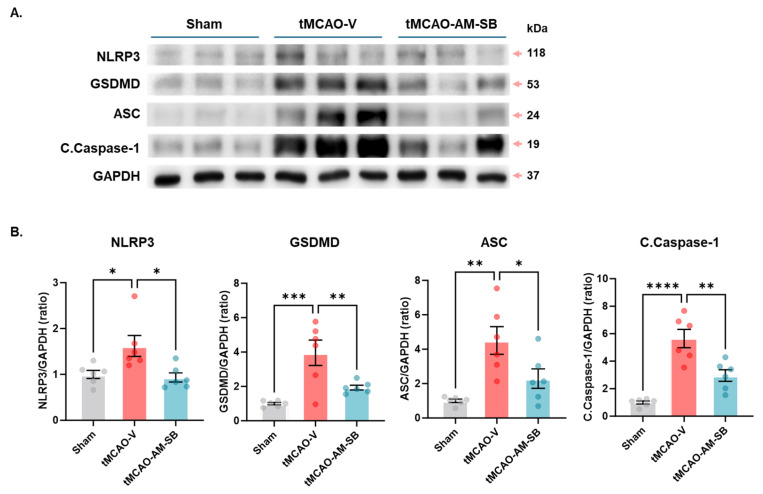
AM-SB reduced pyroptosis in tMCAO model mice. (**A**) Representative Western blots and (**B**) quantification of NLRP3, GSDMD, ASC, and Cleaved Caspase-1 (C. Caspase-1 (p10)) proteins in the brains of tMCAO model mice on day 3 post-tMCAO (Sham, n = 6; tMCAO-V, n = 6; tMCAO-AM-SB, n = 6). Values are expressed as the mean ± SEM. Statistical analysis was performed using one-way ANOVA followed by Dunnett’s multiple comparisons test. * *p* < 0.05, ** *p* < 0.01, *** *p* < 0.001, **** *p* < 0.0001.

In the tMCAO-V group, pyroptosis marker levels were significantly elevated compared to the sham group, indicating an increase in pyroptosis in response to I/R injury: NLRP3, 1.621 ± 0.2273 in tMCAO-V vs. 1 ± 0.08987 in sham, * *p* < 0.05; GSDMD, 3.957 ± 0.7383 in tMCAO-V vs. 1 ± 0.06879 in sham, *** *p* < 0.001; ASC, 4.508 ± 0.8043 in tMCAO-V vs. 1 ± 0.09713 in sham, ** *p* < 0.01; Caspase-1, 5.649 ± 0.6619 in tMCAO-V vs. 1 ± 0.09713 in sham, ** *p* < 0.01 (Figure 4A,B and Figure S5). These data indicate that tMCAO significantly induced pyroptosis, which contributed to cell death and inflammation. Notably, AM-SB treatment also significantly reduced the levels of these pyroptosis markers, indicating its protective effects against pyroptosis-induced cell death: NLRP3 was reduced to 0.9334 ± 0.09937 (* *p* < 0.05); GSDMD was reduced to 1.935 ± 0.1357 (** *p* < 0.01); ASC was reduced to 2.292 ± 0.5674 (* *p* < 0.05); and Caspase-1 was reduced to 2.962 ± 0.4207 (** *p* < 0.01) (Figure 4A,B and Figure S5). Our findings demonstrated that AM-SB treatment significantly modulated the expression of these markers, suggesting its potential to alleviate pyroptosis and mitigate inflammation in the brains of tMCAO mice.

### 2.5. AM-SB Alleviated Neurological Deficits and Brain Atrophy and Improved Motor and Cognitive Functions

**Figure 5:**
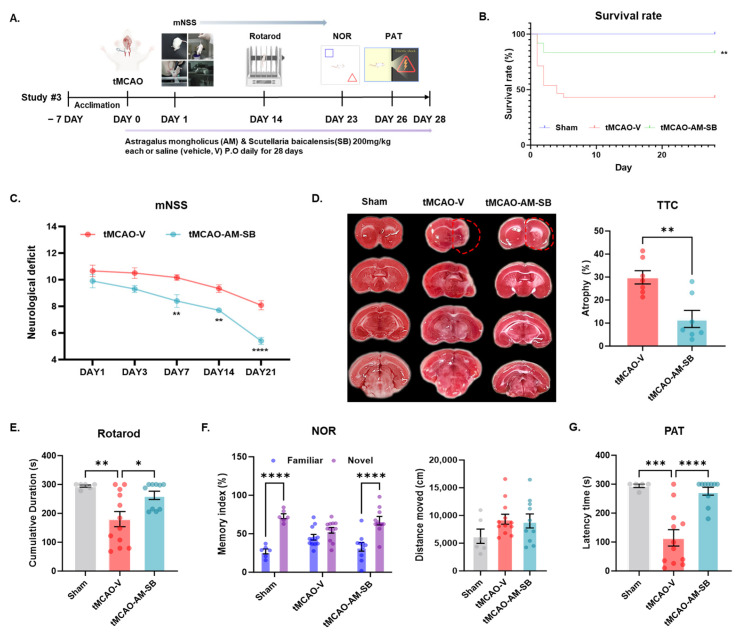
AM-SB administration improved functional outcomes following ischemic stroke. (**A**) Schematic of the experimental timeline. Graphical representation of (**B**) survival rate, (**C**) modified neurological severity scores (mNSSs), (**D**) TTC staining (tMCAO-V, n = 7; tMCAO-AM-SB, n = 7), and the behavioral test results, including the (**E**) Rotarod test, (**F**) Novel Object Recognition (NOR) test, and (**G**) Passive Avoidance test (PAT) over a 28-day period post-tMCAO (Sham, n = 5–6; tMCAO-V, n = 12; tMCAO-AM-SB, n = 10). Values are expressed as the mean ± SEM. Statistical analysis was performed using the log-rank (Mantel-Cox) test for survival rate, two-way ANOVA followed by Šídák’s multiple comparisons test for mNSS and NOR tests, unpaired *t*-test for atrophy, and one-way ANOVA followed by Tukey’s multiple comparisons test for the Rotarod and PAT tests. * *p* < 0.05, ** *p* < 0.01, *** *p* < 0.001, **** *p* < 0.0001.

To evaluate the long-term neuroprotective effects of AM-SB following cerebral ischemic injury, mice were randomly assigned to receive either vehicle or AM-SB daily for 28 days immediately following tMCAO (Figure 5A). Initial body weight measurements indicated a significant reduction in the tMCAO group on day 3 post-tMCAO, followed by recovery (Figure S6A). Notably, AM-SB treatment significantly improved the survival rate of tMCAO mice compared to that of the vehicle-treated group (Figure 5B), suggesting that AM-SB contributes to overall recovery and survival following ischemic injury.

Neurological deficits were evaluated over a 28-day period using the mNSS. Mice treated with AM-SB showed significant improvement in neurological function compared to the vehicle-treated group at multiple time points: day 7: 8.4 ± 1.767 in AM-SB vs. vehicle (** *p* < 0.01); day 14: 7.7 ± 1.633 in AM-SB vs. vehicle (** *p* < 0.01); day 21: 5.4 ± 2.683 in AM-SB vs. vehicle (**** *p* < 0.0001) (Figure 5C). Further, the brain atrophy induced by ischemic stroke was reduced in the AM-SB-treated group (tMCAO-Vehicle, 29.90 ± 2.864; tMCAO-AM-SB, 11.82 ± 3.700, ** *p* < 0.01) (Figure 5D). These findings indicate that AM-SB effectively mitigated the neurological impairments caused by tMCAO.

Motor function recovery was evaluated using the Rotarod test on day 14 after tMCAO. The cumulative duration on the Rotarod was significantly longer in the tMCAO-AM-SB group (262.6 ± 14.39, * *p* < 0.05) compared to the tMCAO-V group (180.2 ± 26.29) (Figure 5E). This result indicates that AM-SB treatment helps to recover motor coordination and endurance in tMCAO mice. Cognitive function was assessed using the Novel Object Recognition (NOR) test on day 23 and the Passive Avoidance test (PAT) on day 26 post-tMCAO. In the NOR test, the memory index, a measure of cognitive function, was significantly higher in the tMCAO-AM-SB group (66.98 ± 7.192, **** *p* < 0.0001) compared to the tMCAO-V group (54.48 ± 5.685) (Figure 5F). Importantly, we found no significant difference in the total distance moved between the groups, indicating that the observed cognitive improvements were not caused by changes in general locomotor activity (Figure S6B). In the PAT, the latency time, reflecting memory retention, was significantly longer in the tMCAO-AM-SB group (275.7 ± 13.71, ** *p* < 0.01) compared to the tMCAO-V group (138.3 ± 28.05) (Figure 5G). These results indicate that AM-SB treatment enhanced memory retention and cognitive function in tMCAO mice.

## 3. Discussion

Ischemic stroke, characterized by brain tissue damage like oxidative stress, inflammation, and cell death resulting from blocked blood flow, remains a major medical challenge with limited effective treatments [24,31,32,33]. In this study, we evaluated the therapeutic potential of *A. mongholicus* (AM) and *S. baicalensis* (SB), EAHM widely used in Eastern medicine, using mouse models that represent the acute, sub-chronic, and chronic phases of stroke. 

Our findings demonstrated the neuroprotective effects of the AM-SB mixture in the PTB model, which mimics acute ischemic injury. The AM-SB treatment mitigated ischemic damage by alleviating thrombus formation and multi-vessel occlusions, resulting in a significant reduction in infarct volume and neurological deficits within the initial 24 h period. These findings indicate that the AM-SB mixture effectively protects brain tissue from acute ischemic damage (Figure 1B). The neuroprotective effects are likely mediated by formononetin and baicalein, the primary active components of AM and SB, respectively, which exhibit strong anti-inflammatory and antioxidant properties [34,35].

In the tMCAO model, designed to study sub-chronic ischemic injury, the AM-SB mixture demonstrated additional therapeutic benefits. Oral administration of the AM-SB, initiated on the day of ischemia induction, significantly reduced infarct volume during the sub-chronic phase, as shown by TTC staining and MRI-T2 imaging on day 3 post-tMCAO. AM-SB also restored cerebral metabolites (Figure 2B–D) and reduced gliosis, as evidenced by immunohistochemistry (Figure 3A–C). These effects align with previous research showing that SB inhibits NF-κB signaling, reducing the expression of inflammatory mediators such as TNF-α and IL-1β [36,37,38,39], while AM polysaccharides similarly attenuate inflammation via NF-κB pathway modulation [40,41,42,43,44,45,46,47]. The significant decrease in inflammatory markers IL-1β and TNF-α in the penumbra region further supports AM-SB’s role in mitigating the inflammatory cascade following ischemic injury (Figure 3C). Baicalein has been shown to suppress neuroinflammation by promoting anti-inflammatory microglia polarization in an ischemic model [48], and formononetin inhibits the NLRP3 inflammasome, a key regulator of the inflammatory process [49]. The AM-SB mixture also demonstrated efficacy in reducing pyroptosis, a form of programmed cell death associated with inflammation. AM-SB significantly reduced the expression of pyroptosis biomarkers, including NLRP3, GSDMD, ASC, and Caspase-1, in the tMCAO model (Figure 4A,B). This indicates that AM-SB effectively mitigates pyroptotic cell death and inflammation, contributing to its neuroprotective effects.

In the chronic phase, AM-SB treatment facilitated long-term recovery of motor and cognitive functions over a 28-day period. The AM-SB-treated group exhibited decreased neurological deficits (Figure 5C), reduced brain atrophy (Figure 5D), improved motor function in the Rotarod test, and enhanced cognitive performance in the Novel Object Recognition and Passive Avoidance tests (Figure 5E–G). These results highlight the ability of AM-SB to not only mitigate acute and sub-chronic ischemic damage but also promote post-stroke functional recovery.

Overall, the findings of this study underscore the potential of AM-SB as a therapeutic strategy for ischemic stroke. By targeting inflammation, oxidative stress, and pyroptosis, the AM-SB mixture offers a multi-faceted approach to stroke management, addressing both immediate and long-term consequences of ischemic injury.

## 4. Materials and Methods

### 4.1. Preparation of the Combination of AM and SB Extracts

The dried roots of *A. mongholicus* Bunge (AM) and *S. baicalensis* Georgi (SB) used in the experiment were purchased from Yaksudang Pharmaceutical Co., Ltd. (Seoul, Republic of Korea). All materials were identified by Professor Donghun Lee, Department of Herbal Medicine, College of Korean Medicine, Gachon University, and voucher specimens (D210511001 and D200915001) were preserved (Figure S7). Dried AM and SB were extracted separately in 30% EtOH under reflux at 85 °C for 3 h. The extracts were then filtered, and the solvent (ethanol and water) was removed under decompressed conditions before lyophilizing the extracts at −80 °C. The extract yields were 14.63% and 28.67%, respectively.

(1) $$Yield\;\left(\%\right)=\frac{Extract\;weight}{Dry\;weight\;of\;plant\;material\;used}*100$$

### 4.2. Analysis of the Combination of AM and SB Extracts Using High-Performance Liquid Chromatography (HPLC)

To analyze the components of the combined AM and SB extracts, high-performance liquid chromatography (HPLC) was performed using an 1100-series HPLC system (Agilent, Santa Clara, CA, USA). The separation was achieved on an Altima HP C18 column (250 mm × 4.6 mm, 5 μm; Alltech, Woodridge, IL, USA) at a column temperature of 30 °C. The mobile phase consisted of (A) 0.1% phosphoric acid in water and (B) acetonitrile (ACN) with the following gradient elution program: 0–10 min, 20–30% B; 10–50 min, 30–50% B; 50–51 min, 50–90% B; 51–53 min, 90–20% B; and 53–55 min, 20–20% B. The flow rate was set at 1.0 mL/min, and the injection volume was 10 μL. The detection wavelengths were 275 nm for baicalein and 250 nm for formononetin. A final concentration of 10 mg/mL for each extract was prepared, diluted with 50% methanol, and sonicated for 20 min before filtration through a 0.45 μm syringe filter (ADVANTEC, Tokyo, Japan). Baicalein and formononetin were quantified in the combination extract with contents of 8.6069 mg/g and 0.2422 mg/g, respectively (Table S1, Figure S1).

### 4.3. Animals

A total of 107 healthy male ICR mice weighing 33 ± 3 g were obtained from DBL Co., Ltd., Eumseong-gun, Chungbuk, Republic of Korea. Mice were housed in a controlled environment at a temperature of 22 ± 2 °C, humidity of 50 ± 10%, and a 12 h light/dark cycle. Mice were allowed unrestricted access to food and water and were given a 1-week adaptation period prior to the experiment. All 107 animal procedures were approved by the Institutional Animal Care and Use Committee of the Lee Gil Ya Cancer and Diabetes Institute, Gachon University (LCDI-2022-0105, LCDI-2024-0079), and the Institutional Animal Care and Use Committee of the Korea Institute of Science and Technology for Eastern Medicine approved the experimental methods (GUIACUC R-2020029).

### 4.4. Photothrombotic (PTB) Mouse Model

Permanent focal cerebral ischemia was induced in 28 male ICR mice via photothrombosis of the cortical microvasculature. Briefly, mice were anesthetized with 3% isoflurane vapor, followed by Rose Bengal (33 mg/kg; Sigma, Burbank, CA, USA, cat. no. 330000) being administered intraperitoneally. The head of each mouse was fixed in a stereotaxic frame (Jeung Do BIO & PLANT Co., Ltd., Nowon-gu, Seoul, Republic of Korea), and the midline of the scalp was incised using a surgical blade (Paragon, Freiburg, Germany, No. 10). A cold light aperture (Carl Zeiss, cat. no. CL6000 LED, Jena, Germany) was positioned 2 mm lateral to the bregma, and the skull was exposed to the illuminator for 15 min. The incision was closed with surgical staples (Jeung Do, MD: 20877/203–1000). During the procedure, the body temperature of the mice was maintained at 37 ± 0.5 °C and monitored for up to 1 h after the administration of Rose Bengal.

To evaluate the neuroprotective effects of AM-SB extract, mice were randomly divided into two groups: a control group and an AM-SB treatment group. The control group received saline, whereas the treatment group received 200 mg/kg of each AM-SB extract orally and was sacrificed the next day.

### 4.5. Transient Middle Cerebral Artery Occlusion (tMCAO) Mouse Model

Focal ischemic stroke was induced in 10-week-old male ICR mice using the transient middle cerebral artery occlusion (tMCAO) method, as previously described. Briefly, the animals were anesthetized with isoflurane (3% for induction and 1.5% for maintenance) and medical oxygen. A heating pad was used to maintain the body temperature within the normal range throughout surgery [50,51,52,53].

After making an incision on the skin of the neck, the right carotid region was exposed, and the external and common carotid arteries were ligated with a 6-0 black silk suture (AILEE Corp., 28 Gaya-daero, 196beon-gil, Seoul, Republic of Korea). A 20 mm length 6-0 silicone-coated monofilament (Doccol Corp., 30 Eisenhower DR, Sharon, MA, USA) was inserted via the external carotid artery to occlude the origin of the middle cerebral artery (MCA) (Figure S2A). Blood flow obstruction during tMCAO was monitored using a laser Doppler flowmeter probe (PeriFlux 6000 system; PERIMED, Järfälla, Sweden) (Figure S2B). After 90 min of occlusion, reperfusion was achieved by gently withdrawing the monofilament, while maintaining the same anesthetic conditions. The control animals underwent sham surgery without suture insertion. Following the procedure, the incision was sutured, and the mice were placed in a warm recovery cage before being returned to their regular cages with free access to food and water.

Seventy-nine mice were randomly assigned to one of three treatment groups: the sham group, which included sham-operated animals that underwent a surgical procedure without MCAO and were treated orally with the control vehicle (0.9% normal saline); the tMCAO vehicle group, which comprised mice subjected to tMCAO and treated orally with the control vehicle; and the tMCAO AM-SB group, which included mice subjected to tMCAO and treated orally with a combination of 200 mg/kg AM-SB extract. The administration volume for all treatments was 5 mL/kg body weight. The first dose was administered orally within one hour of reperfusion in the tMCAO model.

### 4.6. Neurological Deficit Scoring (mNSS) and Survival Rate

Neurological deficits were assessed daily, beginning 24 h after tMCAO, using the mNSS, which ranges from 0 (normal) to 18 (maximal deficit) and evaluates the motor, sensory, balance, and reflex functions. A score of 1 was assigned to the inability to perform a test or the absence of a reflex, with higher scores indicating more severe deficits [54,55,56]. All neurological assessments were conducted by observers blinded to the treatment groups. The survival rate was determined by calculating the percentage of mice that survived throughout the experimental period.

### 4.7. Quantification of Infarction Size

To quantify the infarction size, extracted brain tissue was stained with 2,3,5-Triphenyltetrazolium chloride (TTC) dissolved in 0.9% normal saline [57,58]. The contralateral hemisphere, which was not affected by ischemia, served as a reference for the standard volume of the brain. The extent of infarction in the ipsilateral (damaged) hemisphere was measured, and the infarction size was calculated using the following formula:

Infarction volume (%) = 100 − ((Healthy area of the ipsilateral hemisphere)/ (Area of the contralateral hemisphere)) × 100

### 4.8. Magnetic Resonance Imaging (MRI) and MR Spectroscopy (MRS) Analysis

Magnetic resonance imaging (MRI) was performed on day 3 post-surgery using a Biospec 9.4T MRI system (Bruker BioSpin Corporation, Billerica, MA, USA), equipped with a radiofrequency coil at the Core-Facility for Cell to In Vivo Imaging. The mice were deeply anesthetized with 2.0–2.5% isoflurane and a 1:2 mixture of O_2_ and NO_2_ (250 mL/min) and positioned supine with their heads centered in the coil. Anesthesia was maintained throughout the imaging session, with respiration monitored and body temperature regulated by warm water circulation.

MRI T2-weighted (MRI-T2) images were acquired using the Turbo-RARE sequence, under the following parameters: TE = 17.54 ms, TR = 5000 ms, effective TE = 33 ms, RARE factor = 8, averages = 4, FOV = 15 × 15 mm^2^, matrix size = 150 × 150, and slice thickness = 0.50 mm. These images were used to locate the volume of interest (VOI) for subsequent MR spectroscopy (MRS) in the striatum (8 μL). Localized shimming was performed to correct field inhomogeneity in the VOI, followed by proton MR spectroscopy (1H MRS) using the PRESS sequence, under the following parameters: TE = 17.54 ms, TR = 5000 ms, 320 averages, 2048 data points, spectral width = 4401.41 Hz, and VOI = 2 × 2 × 2 mm^3^. Outer volume and VAPOR water suppressions were incorporated.

The spectral data were analyzed using the LCModel for absolute quantification. Lesion volumes were assessed using T2-weighted images by calculating the ratio of the ischemic region to the contralateral hemisphere.

### 4.9. Behavior Tests

#### 4.9.1. Rotarod Test

To evaluate motor function, mice were initially trained on a Rotarod apparatus (B. S. Technolab Inc., Seoul, Republic of Korea) on the first day. They were allowed to remain on the Rotarod for 5 min at speeds of 10 and 15 rpm. On the second day, motor function was assessed using an accelerating Rotarod, which was increased in speed from 5 to 30 rpm over a 5 min period. The mice were allowed a 30 min rest between trials to minimize exercise stress. The cumulative duration that the mice remained on the Rotarod across the three trials was averaged to determine motor function.

#### 4.9.2. Novel Object Recognition (NOR) Test

The NOR test was subsequently performed three weeks after tMCAO over three consecutive days. On the first day, mice were allowed to freely explore a square area (40 cm × 40 cm × 40 cm) with four black walls for 10 min to habituate them to the environment. On the second day, two identical objects were placed diagonally opposite each other in the arena and the mice were allowed to explore for 10 min. On the third day, one of the objects was replaced with a novel object and the mice were allowed to explore again for 10 min. The activity of the mice was recorded using a charge-coupled device (CCD) camera and analyzed using the Ethovision XT 17.0 motion analysis program.

#### 4.9.3. Passive Avoidance Test

The PAT was conducted over three consecutive days using a Passive Avoidance apparatus (42.5 cm wide × 35.5 cm long, Gemini Passive Avoidance System (San Diego Instruments, San Diego, CA, USA). This apparatus comprises two adjacent chambers, one bright and one dark, connected by a remotely operated gate. On the first day, mice were allowed to explore both chambers for 5 min for acclimatization. On the second day, when a mouse entered the dark chamber through the sliding door, it received a 0.3 mA electric foot shock for 5 s with the gate closed. After 24 h, the latency time for the mice to enter the dark chamber from the bright chamber was measured to assess memory and learning.

### 4.10. Tissue Processing and Molecular Works

#### 4.10.1. Tissue Preparation

The mice were anesthetized with a combination of Zoletil 8.3 mg/kg (Virbac, Carros, France) and Rompun 15.54 mg/kg (Bayer, Leverkusen, Germany) before their brains were removed. For Western blotting (WB) analysis, peri-infarct regions of the cortex from the ipsilateral hemisphere of the brain were dissected following TTC staining and immediately frozen in liquid nitrogen. The contralateral hemispheres were also flash frozen in liquid nitrogen. For immunohistochemistry, brain tissue was fixed in 4% paraformaldehyde at 4 °C for 24 h, and then dehydrated in 30% sucrose solution for 3 days. Dehydrated tissues were embedded in the Optimal Cutting Temperature (OCT) compounds (Sakura, Osaka, Japan) and frozen. Using a cryomicrotome (Cryotome, Thermo Electron Corporation, Waltham, MA, USA), frozen tissues were sectioned into 20 μm thick slices, and subsequently stored in a cryoprotectant solution comprising 30% ethylene glycol and 30% glycerol in Phosphate-Buffered Saline (PBS) at 4 °C.

#### 4.10.2. Western Blot (WB)

Brain tissues were lysed using Radioimmunoprecipitation Assay (RIPA) buffer containing 150 mM NaCl, 1% NP-40, 0.5% sodium deoxycholate, 0.1% sodium dodecyl sulfate (SDS), and 50 mM Tris (pH 8.0) supplemented with protease inhibitors (Roche Applied Science, Mannheim, Germany) and a phosphatase inhibitor cocktail (Sigma-Aldrich, St. Louis, MO, USA). Lysates were incubated on ice for 30 min, then centrifugated at 13,000 rpm for 20 min at 4 °C. The protein concentrations in the lysates were determined using Bradford assay solution (Bio-Rad Laboratories, Inc., Hercules, CA, USA). Equal amounts of protein were loaded onto 8% or 15% SDS–polyacrylamide gel electrophoresis (PAGE) gels. Proteins were transferred onto polyvinylidene difluoride (PVDF) membranes (Merck, Kenilworth, NJ, USA), blocked with 5% skim milk or 3% bovine serum albumin (BSA) in TBS-T at room temperature for 1 h. The membranes were incubated overnight at 4 °C in primary antibodies diluted in TBS-T containing 3% BSA. After washing with TBS-T, the membranes were subsequently incubated with the appropriate secondary antibodies for 1 h at room temperature. Protein bands were finally visualized using Enhanced Chemiluminescence (ECL) detection (ELPISBIO, Daejeon, Republic of Korea) or Immobilon Western Chemiluminescent HRP Substrate (Millipore, Burlington, MA, USA). Band quantification was performed using ImageJ software v1.53t (National Institutes of Health, Bethesda, MD, USA). Each blot was performed at least three times.

The primary antibodies used were as follows: NLRP3 (1:3000, Novus, Centennial, CO, USA), GSDMD (1:3500, Santacruz, Dallas, TX, USA), ASC (1:3000, Santacruz, Dallas), Caspase-1 (1:3000, Santacruz, Dallas), TNF-α (1:3000, Santacruz, Dallas), IL-1β (1:4000, Santacruz, Dallas) and GAPDH (1:10000, Santacruz, Dallas).

#### 4.10.3. Immunohistochemistry (IHC)

To assess changes in gliosis, immunofluorescence analysis was performed as follows: Brain sections were washed in PBS-T (0.4% Triton X-100 in PBS) and blocked with 1% BSA and 3% normal goat serum in 0.4% PBS-T at room temperature for 1 h. The sections were then incubated overnight at 4 °C in PBS-T with primary antibodies against mouse anti-GFAP (1:500, DAKO, Centennial, CO, USA) and rabbit anti-Iba1 (1:500, Sigma, St. Louis, MO, USA). After washing with PBS-T, sections were incubated for 1 h at room temperature with Alexa Fluor 555-conjugated goat anti-rabbit (1:500; Invitrogen, Carlsbad, CA, USA) or Alexa Fluor 488-conjugated goat anti-mouse (1:500; Invitrogen) secondary antibodies. DAPI (Roche, Basel, Switzerland) was also used as a counterstain. Images were captured using a Nikon TS2-S-SM microscope (Nikon Microscopy, Tokyo, Japan), equipped with a Nikon DS-Qi2 camera and analyzed using Image J 1.53t software. The fluorescence intensity in the defined regions of interest was measured and expressed as a percentage.

### 4.11. Statistical Analysis

All statistical analyses and outlier removal were performed using GraphPad Prism version 9.5.1 software (GraphPad Software Inc., San Diego, CA, USA). An outlier significance level of alpha = 0.05 was used. Data are presented as the mean ± standard error of the mean (SEM). Neurological deficits and memory index data, including those from the mNSS and NOR tests, were analyzed using two-way analysis of variance (ANOVA), followed by Šídák’s multiple comparison test. Results from the Rotarod test, PAT, MRS metabolite analysis, IHC, and WB assays were analyzed using one-way ANOVA, followed by Tukey’s multiple comparisons test.

Differences in TTC staining and MRI-T2 image data between the vehicle- and AM-SB-treated groups were assessed using unpaired *t*-tests. Differences in survival rates between the groups were assessed using the log-rank (Mantel–Cox) test. Statistical significance was set at a *p*-value < 0.05, with different levels of significance denoted as follows: * *p* < 0.05, ** *p* < 0.01, *** *p* < 0.001, and **** *p* < 0.0001.

## 5. Conclusions

This study underscores the significant therapeutic potential of the herbal mixture *Astragalus mongholicus* (AM) and *Scutellaria baicalensis* (SB) in mitigating inflammation and pyroptosis via modulation of the NLRP3/Caspase-1/GSDMD pathway in ischemic stroke. Using both photothrombotic (PTB) and transient middle cerebral artery occlusion (tMCAO) mouse models, AM-SB administration demonstrated neuroprotective effects, including reductions in infarct volume, attenuation of gliosis, and suppression of key pyroptosis markers such as NLRP3, GSDMD, and Caspase-1. These findings indicate that AM-SB effectively addresses both the acute and sub-chronic phases of ischemic injury, reducing secondary brain damage and promoting long-term functional recovery. Behavioral assessments, including the modified Neurological Severity Scores (mNSSs), Rotarod, Novel Object Recognition (NOR), and Passive Avoidance test (PAT), revealed substantial improvements in motor and cognitive functions. Furthermore, AM-SB displayed broad-spectrum efficacy by restoring metabolic balance and reducing inflammatory cytokines such as TNF-α and IL-1β. Mechanistically, the active components of AM (formononetin) and SB (baicalein) contributed to the reduction in gliosis, inflammation, and pyroptosis, emphasizing their neuroprotective potential.

Despite these promising results, certain limitations should be acknowledged. The study did not evaluate the integration of AM-SB with thrombolytic agents like tPA, the current standard of care for ischemic stroke, limiting its direct clinical applicability [59]. Additionally, the use of young, healthy animal models does not account for common stroke-related comorbidities, such as hypertension and diabetes, which may significantly impact treatment outcomes. Future research should consider incorporating aged animal models with relevant comorbidities to better replicate clinical scenarios and evaluate the combined efficacy of AM-SB with existing therapies.

## Data Availability

The data supporting the findings of this study are available from the corresponding author upon reasonable request.

## Supplementary Materials

The following supporting information can be downloaded at: https://www.mdpi.com/article/10.3390/ijms26020501/s1.
