# Supplementary material for: Astragalus mongholicus and Scutellaria baicalensis Extracts Mixture Target Pyroptosis in Ischemic Stroke via the NLRP3 Pathway
Source: Int J Mol Sci. 2025 Jan 9;26(2):501. doi: 10.3390/ijms26020501 (PMC11765050; doi:10.3390/ijms26020501)
Supplement: Supplementary file 1 [file ijms-26-00501-s001.zip › ijms-3369487-supplementary.pdf]

Table S1. Condition for HPLC analysis

**Column**	Altima HP C18 column (250 mm × 4.6 mm, 5 μm ; Alltech (IL, USA)
**Mobile phase**	(A) 0.1% Phosphoric acid, (B) ACN<br>0-10 min, 20-30%; 10-50 min, 30-50%; 50-51 min, 50-90%; 51-53 min, 90-20%; 53-55 min, 20-20% (B)
**Flow rate**	1.0 mL/min
**Injection volume**	10 μL
**Detection wavelength**	275 nm, 250 nm
**Temperature**	30 °C

Supplementary Figures

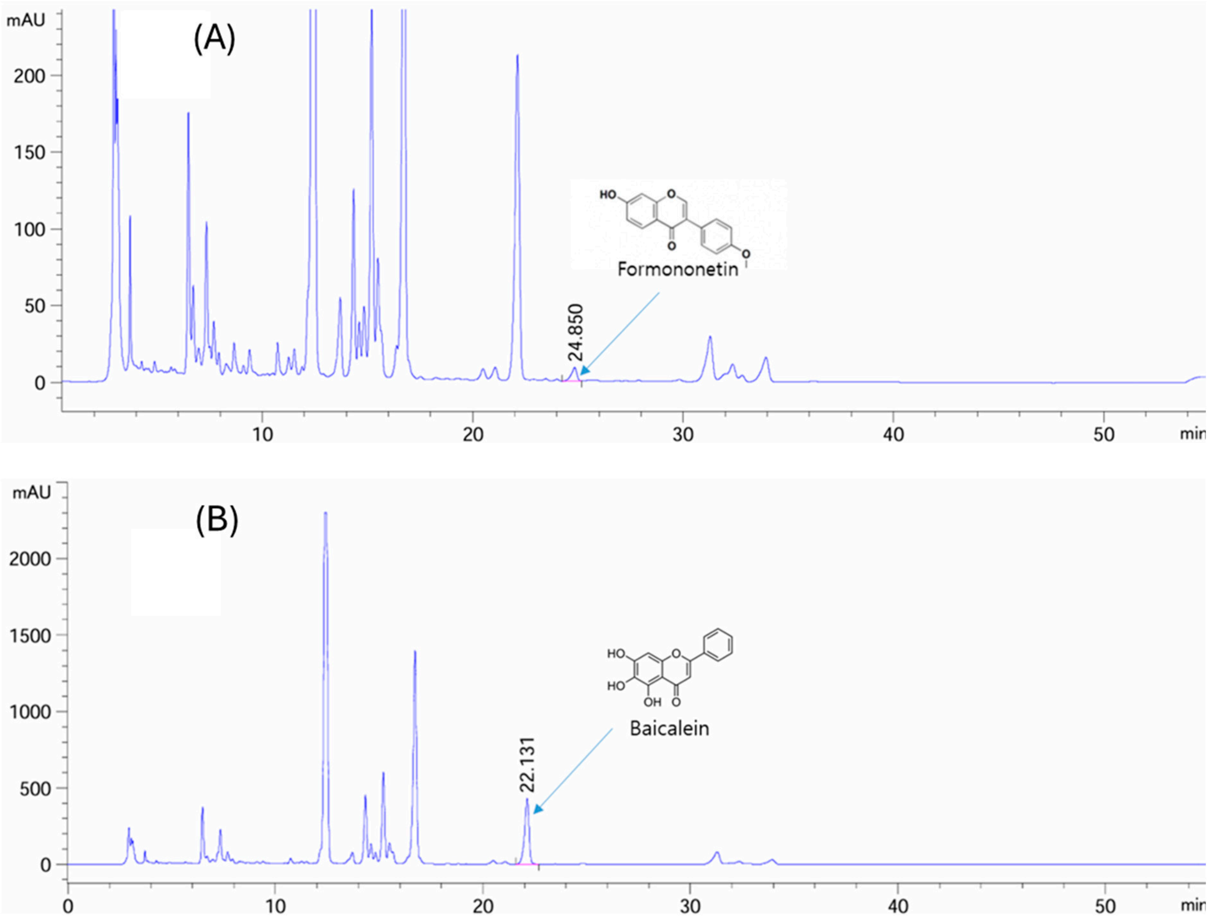

Figure S1. HPLC analysis

The high-performance liquid chromatography chromatogram of the combination extract at 250 nm shows formononetin with a retention time of 24.850 minutes (A), and at 275 nm, baicalein with a retention time of 22.131 minutes (B). The x-axis represents the retention time, and the y-axis represents the absorbance units. An Altima HP C18 column (250 mm × 4.6 mm, 5 μm; Alltech, IL, USA) was used for chromatographic separation at 30 °C.

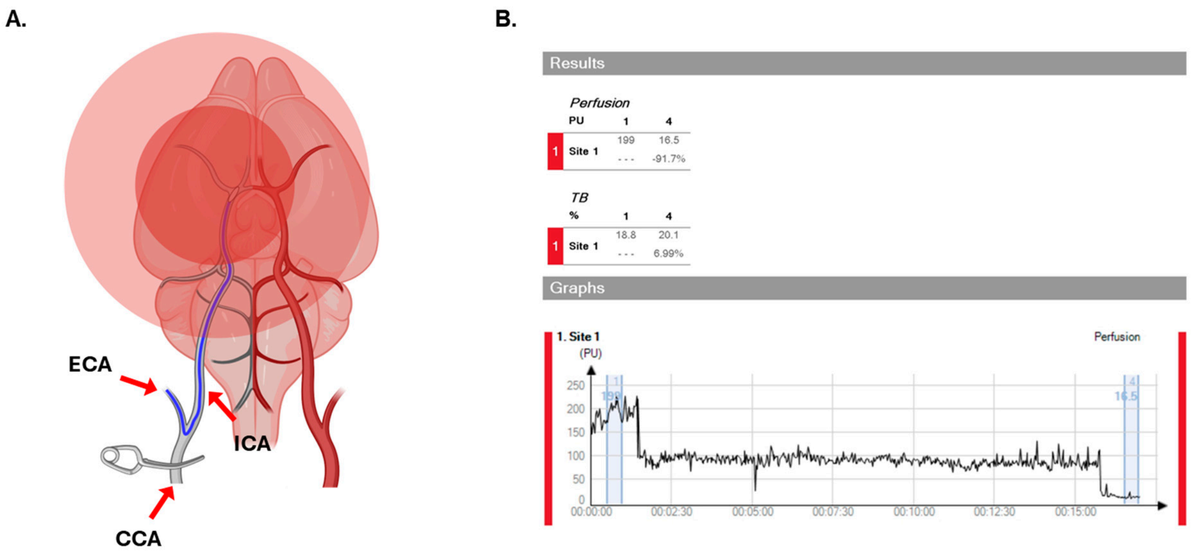

Figure S2. Modeling of tMCAO, Blood flow tracking

(A) Illustration of the transient middle cerebral artery occlusion (tMCAO) procedure and (B) monitoring image showing cerebral blood flow post-occlusion and reperfusion using laser doppler flow meter.

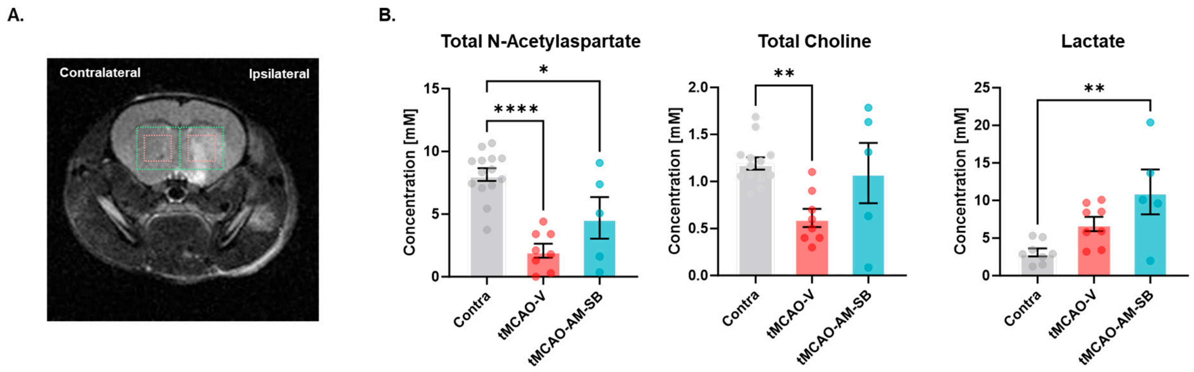

Figure S3. T2-weighted MRI and MRS data in tMCAO Mouse Model.

(A) A T2-weighted MRI image showing the overlaid voxel positions for MRS acquisition (red boxes) and the regions used for shimming (green boxes) in a tMCAO mouse model. (B) Quantitative graph depicting cerebral metabolites levels of total N-Acetylaspartate, total choline and lactate in tMCAO mice on day 3 post-tMCAO. (Contralateral, n=8-14; tMCAO-V, n=8; tMCAO-SB-AM, n=5). Values are expressed as the mean ± SEM. Statistical analysis among the three groups was performed using one-way ANOVA following by Dunnett's multiple comparisons test. ***p*<0.01.

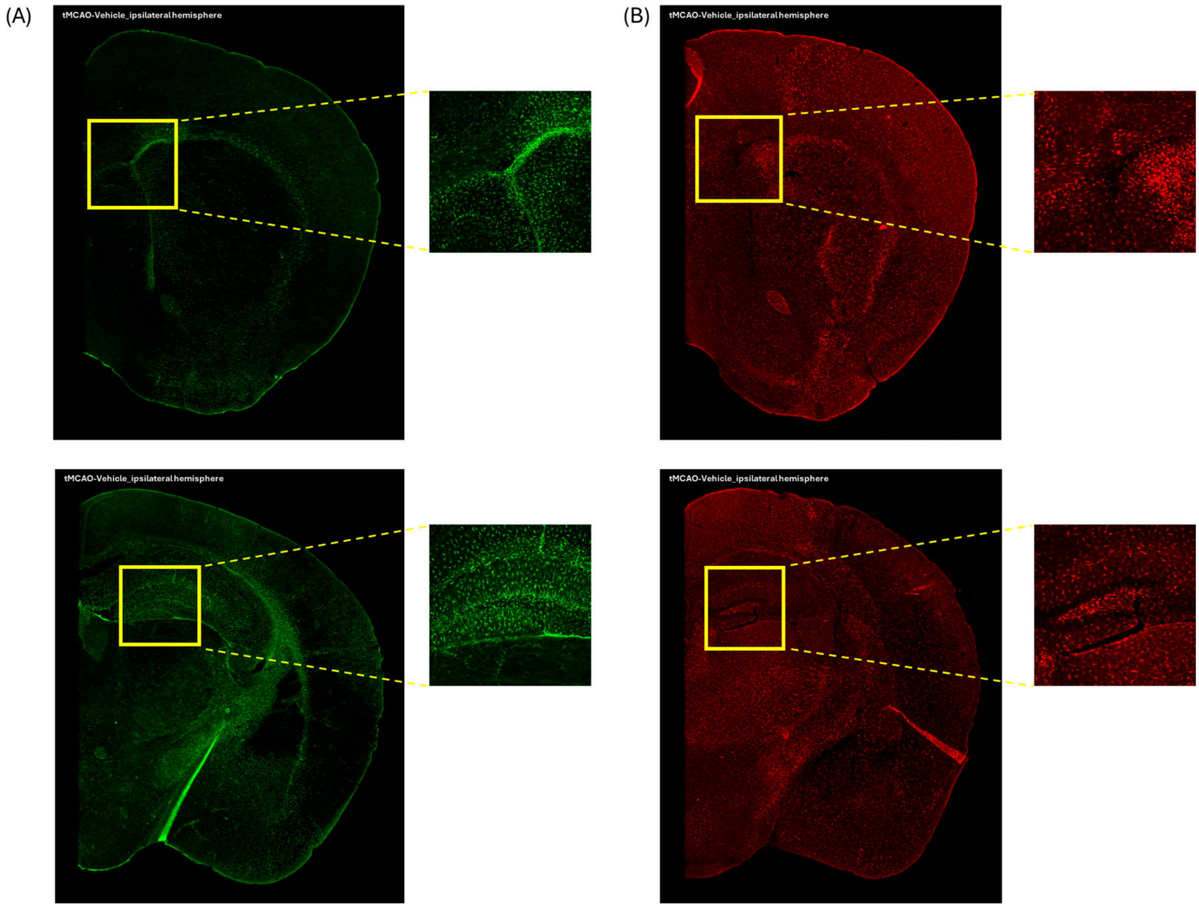

Figure S4. Ipsilateral hemisphere of tMCAO vehicle mouse brain with gliosis marker

(A) ipsilateral hemisphere of tMCAO vehicle mouse brain with GFAP+ cells. (B) ipsilateral hemisphere of tMCAO vehicle mouse brain with Iba-1+ cells.

Figure. 2C

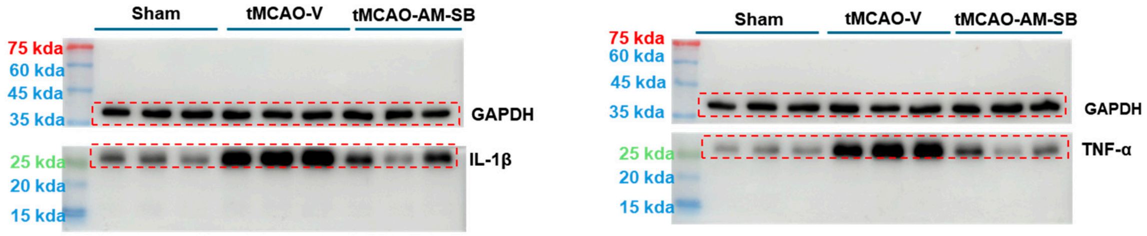

Figure. 3A

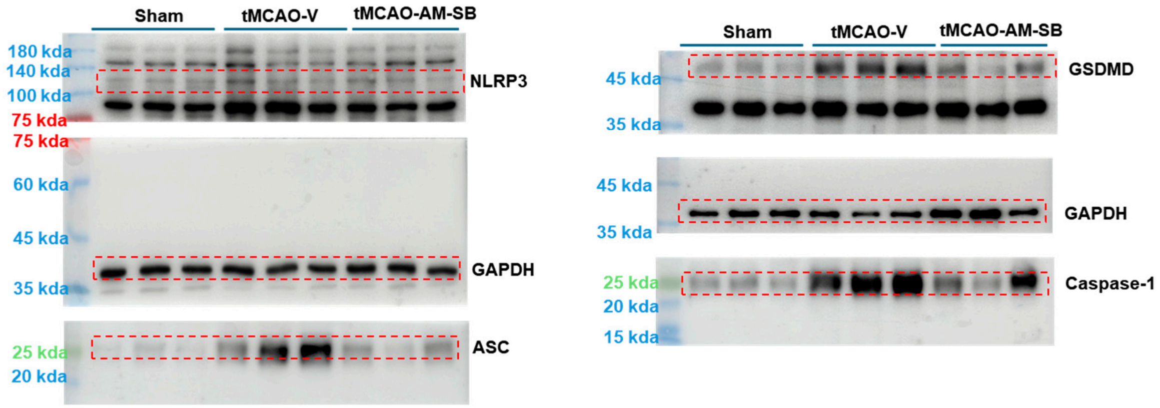

Figure S5. Full blot Corresponding to figure 2C and figure 3A.

This figure presents the full Western blots for IL-1β, TNF-α and GAPDH, corresponding to the data shown in Figures 2C and 3A.

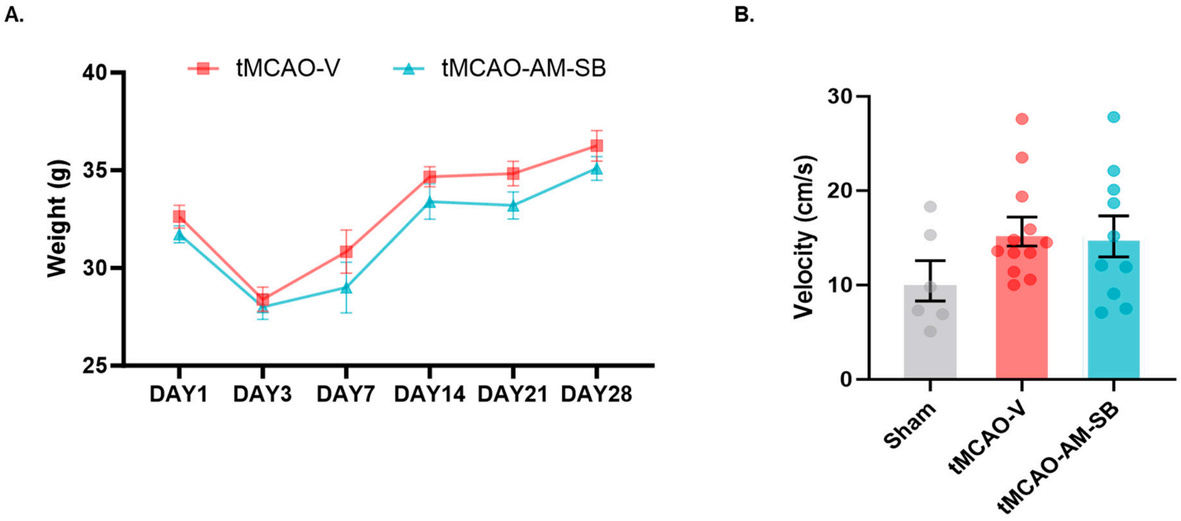

Figure S6. Behavioral Analysis and Performance Metrics in Experimental Mice.

(A) Body weights of mice from the two experimental groups used in the behavioral tests presented in Figure 4. (B) Velocity measurements during the novel object recognition (NOR) test, showing the moving speed among the three groups.

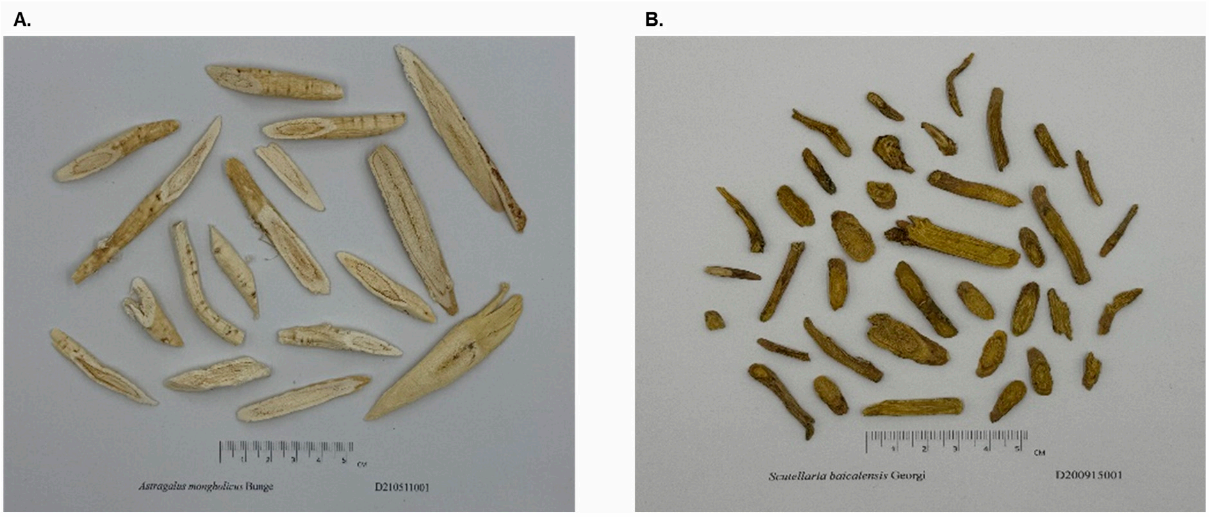

Figure S7. Picture of *Astragalus mongholicus* Bunge (A) and *Scutellaria baicalenesis* Georgi (B)
